# Effects of Transplanted Heparin-Poloxamer Hydrogel Combining Dental Pulp Stem Cells and bFGF on Spinal Cord Injury Repair

**DOI:** 10.1155/2018/2398521

**Published:** 2018-03-27

**Authors:** Lihua Luo, Abdullkhaleg Ali Albashari, Xiaoyan Wang, Ling Jin, Yanni Zhang, Lina Zheng, Jianjian Xia, Helin Xu, Yingzheng Zhao, Jian Xiao, Yan He, Qingsong Ye

**Affiliations:** ^1^WMU-UQ Research Group for Regenerative Medicine, Institute of Stem Cells and Tissue Engineering, School of Stomatology, Wenzhou Medical University, Wenzhou 325035, China; ^2^School of Pharmaceutical Sciences, Wenzhou Medical University, Wenzhou 325035, China; ^3^School of Dentistry, The University of Queensland, 288 Herston Road, Brisbane, QLD 4006, Australia

## Abstract

Spinal cord injury (SCI) is one of serious traumatic diseases of the central nervous system and has no effective treatment because of its complicated pathophysiology. Tissue engineering strategy which contains scaffolds, cells, and growth factors can provide a promising treatment for SCI. Hydrogel that has 3D network structure and biomimetic microenvironment can support cellular growth and embed biological macromolecules for sustaining release. Dental pulp stem cells (DPSCs), derived from cranial neural crest, possess mesenchymal stem cell (MSC) characteristics and have an ability to provide neuroprotective and neurotrophic properties for SCI treatment. Basic fibroblast growth factor (bFGF) is able to promote cell survival and proliferation and also has beneficial effect on neural regeneration and functional recovery after SCI. Herein, a thermosensitive heparin-poloxamer (HP) hydrogel containing DPSCs and bFGF was prepared, and the effects of HP-bFGF-DPSCs on neuron restoration after SCI were evaluated by functional recovery tests, western blotting, magnetic resonance imaging (MRI), histology evaluation, and immunohistochemistry. The results suggested that transplanted HP hydrogel containing DPSCs and bFGF had a significant impact on spinal cord repair and regeneration and may provide a promising strategy for neuron repair, functional recovery, and tissue regeneration after SCI.

## 1. Introduction

Spinal cord injury (SCI) is a common disease of the central nervous system, resulting in partial or complete loss of motor and sensory functions [1]. The pathological process of SCI can be divided into two phases: the first phase contains a primary mechanical damage of spinal cord which leads to direct damage and loss of axons, neuronal cells, and blood vessels and the second phase includes a secondary injury of neuroinflammatory response which results in excitotoxicity, blood-brain barrier disruption, oxidative stress, and apoptosis [2, 3]. This complicated pathophysiology prevents spinal cord tissue from regeneration and repair. Meanwhile, functional recovery after SCI by conventional therapies has been rarely promoted to a satisfactory level due to the limitation of necessary precursor cells which are crucial in neuron renewal and glial cell regeneration [4, 5]. Therefore, the development of a promising strategy to promote functional recovery after SCI remains to be a significant challenge.

There are some studies suggesting that stem cell transplantation will provide an effective approach for SCI treatment due to their neural differentiation potential. This potential could offer new neural cells to replace dying cells and produce numerous trophic factors, for example, nerve growth factor (NGF), brain-derived neurotrophic factor (BDNF), glial cell line-derived neurotrophic factor (GDNF), and neurotrophin 3 (NT-3), in order to promote neural survival and regeneration [6]. Dental stem cells (DSCs), for instance, dental pulp stem cells (DPSCs), stem cells from human exfoliated deciduous teeth (SHEDs), stem cells from the apical papilla (SCAPs), dental follicle stem cells (DFSCs), and periodontal ligament stem cells (PDLSCs), are considered to be an attractive source of mesenchymal stem cells (MSCs) and exhibit stem cell characteristics such as self-renewal and multilineage differentiation potential [7, 8]. DPSCs, the first dental-related stem cells, were isolated from the third molars in 2000 by Dr. Gronthos [9]. DPSCs have been found to possess the characteristics of MSCs such as multidifferentiation potential and neuroprotective and immunomodulatory properties and to express MSC-like markers such as CD73, CD90, CD105, CD146, CD166, and STRO-1 [10, 11]. Furthermore, originating from the cranial neural crest, DPSCs can also express neural markers such as nestin, *β*-tubulin III, glial fibrillary acidic protein (GFAP), and microtubule-associated protein-2 (MAP-2) [6, 12, 13]. Studies suggest that when induced, DPSCs can differentiate into neuronal-like and oligodendrocyte-like cells, which results in axonal regeneration and repair after SCI [14, 15]. Evidence also demonstrates that DPSC transplantation can promote motor functional recovery by secreting BDNF, GDNF, and NT-3 after SCI. Therefore, DPSCs have the ability to provide beneficial strategy for SCI treatment due to their neuronal differentiation potential and neuroprotective and neurotrophic properties [16, 17]. However, it is unlikely to achieve a complete restoration of neuron function after SCI by DPSC-alone strategy because it is difficult to guarantee an effective cellular density and growth in injured site. Recently, researchers and clinicians propose that the tissue engineering technology including scaffolds, cells, and growth factors will provide a promising strategy for SCI treatment [18, 19].

Human basic fibroblast growth factor (bFGF) is a member of the fibroblast growth factor family, having the ability to mediate cell proliferation and survival in vitro [20]. Besides, bFGF is highly expressed in nervous system and it has been confirmed that bFGF can provide neuroprotection for injured axons and neurons facilitating functional recovery after SCI [19, 21]. However, as a biological macromolecular protein, it is difficult for bFGF to pass through the blood spinal cord barrier, and bFGF is disadvantaged by its rapid diffusion and short half-life time [22]. To overcome these shortcomings, an in situ delivery system should be designed to locally diffuse bFGF in injured site of SCI.

Biomaterial scaffold is one of the key components of tissue engineering to provide a platform for cell adhesion and transplantation and to permit delivery of growth factors to ensure cell survival and proliferation [23]. In addition, some studies indicate that biomaterial scaffold interacting with seeded cells has an ability to modulate cellular functions and behaviors. For instance, substrate stiffness, matrix topology, structure, mechanical force, and biochemical property of biomaterial scaffolds form different tissue-specific microenvironments regulating cell growth, proliferation, migration, and differentiation [24–27]. Therefore, ideal scaffolds should have low/nontoxicity, good cytocompatibility, and tissue compatibility, structural stability and mimic 3D biological microenvironment [23]. Recently, hydrogels have been recognised as attractive scaffolds for tissue engineering due to similar 3D network structure to the natural extracellular matrix, the ability to accommodate cells and to deliver bioactive molecules, and the capability to maintain the structure and porosity of scaffold [28–30].

In this work, we designed a novel thermosensitive heparin-poloxamer (HP) hydrogel containing bFGF and DPSCs, which could be delivered to injured site in spinal cord in order to ensure high density of DPSCs and sustained effect of bFGF during recovery. Our previous studies indicated that HP had a characteristic of controlled phase transition by the variation of temperature, presenting a solution state at 4°C and becoming a hydrogel state at human body temperature [31]. Moreover, HP has a high affinity to growth factors (GFs) such as nerve growth factor (NGF) and acidic fibroblast growth factor (aFGF) and is also able to protect GFs from degradation of protease [32, 33]. In addition, HP hydrogel shows a 3D porous structure and has great cytocompatibility [30, 34]. Thus, in this study, we used HP as the engineered tissue scaffold and transplanted HP hydrogel containing bFGF and DPSCs into the injured site of the spinal cord in order to investigate the effects of DPSCs combined with bFGF on neural regeneration and functional recovery after SCI.

## 2. Materials and Methods

### 2.1. Isolation and Culture of DPSCs

Impacted third molars with no caries, periodontal disease, or periapical disease were collected from healthy volunteers (18–30 years old) at the Department of Oral and Maxillofacial Surgery, Stomatological Hospital of Wenzhou Medical University, Wenzhou, China. Tooth surfaces were sterilized by 75% alcohol and the dental pulp tissues were removed by dental handpiece, then cut into small pieces (approximately 1 mm × 1 mm × 1 mm) and washed three times with 2.5% antibiotics of phosphate-buffered saline (PBS). The pulp tissues were put into 1.5 mL EP tube and digested with 3 mg/mL collagenase type I (Gibco, USA) and 4 mg/mL dispase (Sigma, Germany) for 30 minutes at 37°C. Then cell suspension and pulp tissue were cultured at 37°C in 5% CO_2_ humidified atmosphere with *α*-modified Eagle's medium (*α*-MEM, Gibco, USA) supplemented with 20% fetal bovine serum (FBS, Gibco, USA), 100 U/mL penicillin, and 100 *μ*g/mL streptomycin (Gibco, USA). The culture medium was replaced after the first 5 days culture, and then the culture medium was replaced every 3 days routinely.

### 2.2. Flow Cytometry

The characteristics of DPSCs were identified by flow cytometry and the identification procedure of DPSCs was described as previous method [35]. Briefly, after 90% confluence, the second passage of DPSCs was characterized by flow cytometry using the antibodies of human CD73 (BD Pharmingen™, USA), CD90 (BD Pharmingen, USA), CD166 (BD Pharmingen, USA), CD19 (BioLegend, USA), CD14 (BioLegend, USA), and HLA-DR (BioLegend, USA) according to standard protocols. The data were evaluated with CytoFLEX flow cytometers (Beckman Coulter, California, USA).

### 2.3. Multilineage Differentiation

The multidifferentiation potential of DPSCs was analyzed by osteogenic, adipogenic, chondrogenic, and neurogenic differentiations. The process of differentiation was described as follows:

Osteogenic differentiation: After 60%–70% confluence, the culture medium was carefully aspirated from each well and replaced with 2 mL of OriCell TM mesenchymal stem cell osteogenic differentiation medium (Cyagen, USA), and the medium was changed every 3 days. After 2 weeks of differentiation, cells were fixed with 4% formaldehyde for 30 minutes and stained with alizarin red S for 5 minutes. Finally, the cells were visualized and analyzed under light microscope (TS100, Nikon, Japan).

Adipogenic differentiation: When cells were approximately 100% confluent or postconfluent, growth medium was carefully aspirated off and OriCell TM mesenchymal stem cells adipogenic differentiation medium (Cyagen, USA) was added according to the manufacturers' instructions. By the end of differentiation, cells were fixed with 4% formaldehyde for 30 minutes and stained with oil red O for 30 minutes. The cells were visualized and analyzed by light microscope (TS100, Nikon, Japan).

Chondrogenic differentiation: DPSCs were collected with the density of 2.5 × 10^5^ cells per well and centrifuged at 1000 rpm for 5 minutes at room temperature. Then the pellets were incubated with chondrogenic medium at 37°C, 5% CO_2_ humidified atmosphere. The chondrogenic medium was changed every 3 days. After 30 days, chondrogenic pellets were harvested, fixed with 4% formaldehyde and stained with Alcian blue, and then visualized and analyzed by light microscope (TS100, Nikon, Japan).

Neurogenic differentiation: DPSCs were plated onto cover slips in 6-well plates with the density of 4 × 10^4^ cells per well and incubated at 37°C in 5% CO_2_ humidified atmosphere. After 1 day, the cell culture medium was changed to neuronal induced medium with serum-free DMEM-high glucose containing 10^−7^ M dexamethasone, 50 *μ*g/mL ascorbic acid-2-phosphate, 50 *μ*M indomethacin, 10 *μ*g/mL insulin, and 0.45 mM 3-isobutyl-1-methyl-xanthine for 6 days, and then the neuronal induced medium was changed every 3 days. Finally, the cells were evaluated by immunofluorescence staining with Nestin (1 : 1000, *Sigma-Aldrich,* USA), NeuN (1 : 200, Thermo Fisher, USA), GFAP (1 : 200, *Sigma-Aldrich,* USA), and *β*-tubulin III (1 : 2000, *Sigma-Aldrich,* USA).

### 2.4. CCK-8 Assay

To determine the efficiency of bFGF affecting DPSCs in vitro, cell proliferation was evaluated by CCK-8 assay (Dojindo, Kumamoto, Japan) as the following method [36]. Human basic fibroblast growth factor (bFGF) was synthesized and provided by the Key Laboratory of Biotechnology and Pharmaceutical Engineering, Wenzhou Medical University. DPSCs were plated into 96-well plates with the density of 2.0 × 10^3^ cells per well and cultured in *α*-MEM supplemented with 10% FBS, 100 U/mL penicillin, and 100 *μ*g/mL streptomycin, which was used as the control group. The experimental group was the culture medium containing bFGF and the final concentration of bFGF was set at 20 ng/mL [37]. The cell culture medium was changed every other day. After 1, 3, 4, 5, 6, and 7 days' incubation, 10 *μ*L of CCK-8 solution was added into each well and incubated for another 1 hour in 5% CO_2_ at 37°C. The OD values were measured photometrically at 450 nm by an absorbance microplate reader (Varioskan LUX, Thermo Fisher, USA).

### 2.5. Fabrication of HP-bFGF and HP-bFGF-DPSC Hydrogels

Poloxamer 407 was obtained from Badische Anilin Soda Fabrik Ga (Shanghai, China). The preparation of heparin-poloxamer (HP) was described in our previous work as follows [31]. Firstly, poloxamer 407 and diaminoethylene solutions were used to prepare monoamine-terminated poloxamer (MATP). Then, MATP was reacted with heparin salt by 1-ethyl-3-(3-dimethylaminopropyl)-carbodiimide (EDC) and N-hydroxysuccinimide (NHS) in 4-morpholine ethane sulfonic acid (MES) buffer for 1 day in order to form the amide bonds through the amine groups of poloxamer 407 coupling with the carboxyl ones of heparin. Finally, the mixture was dialyzed with deionized water about 72 hours and lyophilized by freeze dryer in order to obtain the final product.

HP-bFGF hydrogel was prepared according to the cold method as previously described [38]. Briefly, lyophilized HP powder was mixed with bFGF solution at 4°C under modest stirring for 2 hours, and then the mixture was stored at 4°C overnight to obtain a transparent solution, resulting in HP loaded with bFGF.

For the preparation of HP/HP-bFGF loaded with DPSC hydrogel, DPSCs were detached and collected by centrifuging at 1000 rpm for 5 minutes and resuspended in complete *α*-MEM medium. Then the DPSC suspensions were added into HP/HP-bFGF solution and thoroughly mixed at 4°C, kept at 37°C, 5% CO_2_ incubator to obtain HP-DPSC and HP-bFGF-DPSC hydrogels.

### 2.6. Morphology of HP and HP-bFGF Hydrogels

The morphology of HP and HP-bFGF hydrogels were observed by scanning electron microscope (SEM, H-7500, Hitachi, Japan) with 15 kV as the accelerating voltage. HP and HP-bFGF hydrogels were wiped on copper meshes and frozen in liquid nitrogen. Then, the hydrogels were dried at critical point by vacuum freeze dryer for 48 hours. The surfaces of specimens were sputter-coated with gold for SEM observation. The average apparent pore size (*dpore*) was measured from the SEM images by a high-resolution imaging treatment system (HLPAS-1000, Wenzhou Medical University, Wenzhou, China).

### 2.7. Live/Dead Assay

The percentage of viable DPSCs in hydrogels was evaluated by a Live/Dead Viability/Cytotoxicity Kit (Invitrogen, CA, USA) according to the manufacturer's instructions. After DPSCs cocultured with HP and HP-bFGF hydrogels for 48 hours, the samples were incubated with reagents at 37°C for 15 minutes and then observed by fluorescence microscope (Eclipse 80i, Nikon, Japan).

### 2.8. Animal Model of SCI and HP-bFGF-DPSC Hydrogels Application

72 Sprague–Dawley female rats (200–250 g) were purchased from the Animal Center of Chinese Academy of Science (Shanghai, China) and housed in the animal care facility for 14 days prior to surgery. All experiments were performed according to the Guide of Chinese National Institutes of Health and the Animal Care and Use Committee of Wenzhou Medical University. The experiments were divided into 6 groups, including HP, HP-bFGF, HP-DPSCs, HP-bFGF-DPSCs, SCI, and sham control groups. The working concentration of bFGF was set at 3 *μ*g/*μ*L [39]. Animals were anesthetized by intraperitoneal administration of 10% chloralic hydras at a dose of 3.5 mL per kg body weight. After anesthesia, back hair was shaved and the skin was sanitized with 70% alcohol solution. After incision extended along the middle of back and exposed the vertebral column, a laminectomy was performed at T9 segmental level vertebrae. The spinal cord was completely exposed and a moderate injury was created by a vascular clip clipping for 2 minutes (30 g forces, Oscar, China). After SCI, 10 *μ*L (containing 3 *μ*g/*μ*L bFGF wherever applicable) of HP, HP-bFGF, HP-DPSC, and HP-bFGF-DPSC hydrogels was in situ injected by a microsyringe. Animals in SCI model group received the same surgical procedures and were injected with sterile saline solutions (10 *μ*L per animal). The sham control group received the same surgical procedures but the spinal cord was not injured by the vascular clip. Postoperative animals were conventionally housed and the bladder was manually emptied twice a day.

### 2.9. Functional Recovery Analysis

The functional recovery of all animals was evaluated by Basso, Beattie, and Bresnahan (BBB) scoring method, inclined plane test, Reuter scoring method, and footprint test as described previously [29, 40, 41]. The BBB scoring method, ranging from 0 (no movement) to 21 (normal gait), was performed to assess motor functional improvement at day 1, 3, 7, 14, 21, and 28. For the inclined plane test, all animals were performed at the same time points. The maximum angle of the inclined plane on which the animal stayed for 5 seconds was recorded for each rat, and the average score was used for each group. The Reuter scoring method, ranging from 0 (normal) to 11 (no sensory), was performed to assess the sensory functional recovery at the same time as above, including stretch reflex, pain retraction reflex, back feeling, muscle tension, and muscle strength. The footprint assay was performed at day 28 by staining the hind paws of animals with red dye. All functional assays were recorded by two independent observers blinded to the experimental protocol.

### 2.10. Western Blot Analysis

For western blot analysis of in vivo proteins collected at day 7 and 21, the spinal cord segments (0.5 cm in length) at the contusion epicenter were dissected and stored at –80°C as soon as possible. According to protein extraction, the spinal cord tissues were homogenized in modified RIPA buffer including protease inhibitor cocktail (10 *μ*L/mL, GE Healthcare Biosciences, PA, USA) and centrifuged at 12,000 rpm. Supernatant was collected for protein analysis. The extracts were quantified with bicinchoninic acid reagents (BCA, Thermo Scientific Pierce, USA). Proteins (80 *μ*g) were added to electrophoresis in 10% SDS-PAGE gels and then transferred onto the poly(vinylidene difluoride) membranes (PVDF, Millipore, Germany). The membranes were blocked with 5% (*w*/*v*) milk (BD, USA) in tris-buffered saline with 0.05% Tween-20 (TBST) for 90 minutes and incubated with the following primary antibodies at 4°C for 16 hours: Bcl-2 (1 : 300, Santa, USA), Bax (1 : 1000, CST, USA), Caspase-3 (1 : 1000, Abcam, Britain), MBP (1 : 300, Santa, USA), and GAP-43 (1 : 1000, Abcam, Britain). Then the horseradish peroxidase-conjugated secondary antibodies were added to the membranes for another 1 hour at room temperature. Detection of target proteins were performed by ChemiDoc XRS^+^ imaging system (Bio-Rad). All tests were performed in triplicate.

### 2.11. Magnetic Resonance Imaging (MRI), Histology Evaluation, and Immunohistochemistry Analysis

After 28 days, animals were anesthetized and analyzed by MRI in order to observe the regeneration of spinal cord. All animal experiments were performed using GE Signa HDxT 3.0T superconducting MRI imager (GE Medical Systems, USA) in the Second Affiliated Hospital, Wenzhou Medical University. High-resolution sagittal images were obtained from each animal using a spin-echo T2-weighted MRI sequence (TR/TE: 2560/92 ms, FOV: 9 cm, acquisition matrix: 320 × 256, NEX: 4.0, slice thickness: 1.5 mm, band width: 41.67 kHz). During MRI scanning process, animals were put in a thermostat-heated cradle to maintain the body temperature at 37°C.

For histology evaluation after 28 days, animals were euthanized and tissue of interest was perfused with 4% paraformaldehyde in 0.01 M PBS (pH = 7.4). Sections of spinal cord at T8–T10 were fixed with 4% paraformaldehyde for 6 hours, dehydrated with increasing concentrations of ethanol and embedded in paraffin, cut into segments with thickness of 4–6 mm, and stained with hematoxylin-eosin (HE). HE-stained tissue samples at section T8–T10 were observed by light microscope (TS100, Nikon, Japan). As to immunohistochemistry, the sections were treated with primary antibody of GAP-43 (1 : 300, Abcam, Britain) overnight at 4°C and incubated with horseradish peroxidase-conjugated secondary antibodies for another 2 hours at 37°C. Then the sections were blocked with 3,3′-diaminobenzidine (DAB). All sections were observed by fluorescence microscope (Eclipse 80i, Nikon, Japan).

### 2.12. Statistical Analysis

**Figure 1:**
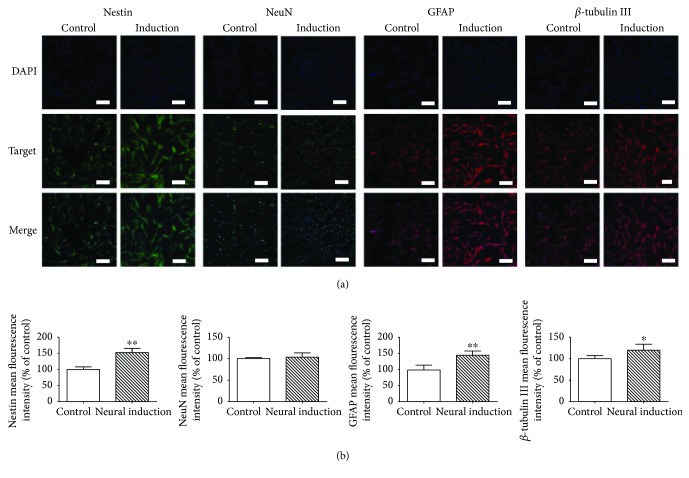
The neurogenic differentiation potential of DPSCs. (a) The expressions of Nestin, NeuN, GFAP, and *β*-tubulin III. Scale bar: 200 *μ*m. (b) Quantification of the fluorescence intensity of Nestin, NeuN, GFAP, and *β*-tubulin III. ^∗^ *P* < 0.05, ^∗∗^ *P* < 0.01 versus the control group.

**Figure 2:**
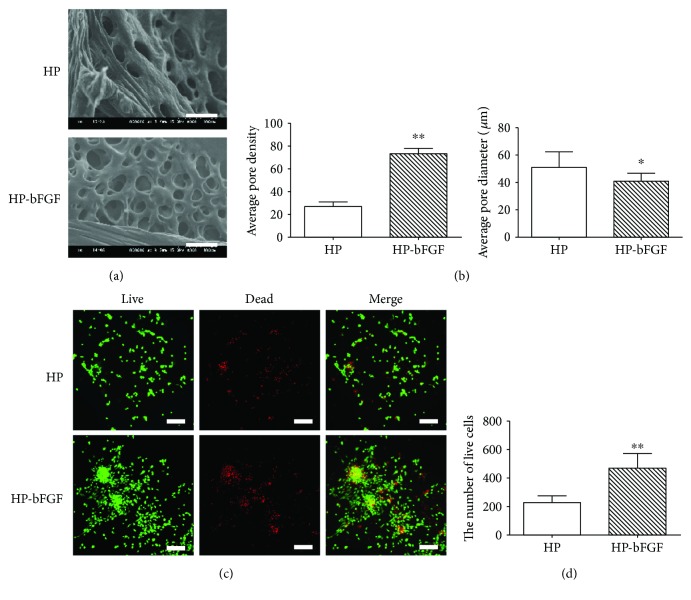
Morphology of HP and HP-bFGF hydrogels and cytocompatibility of hydrogels with DPSCs. (a) SEM images of HP and HP-bFGF hydrogel. Scale bar: 100 *μ*m. (b) Quantification of pore density and pore size. (c) Live/Dead staining of cells in HP and HP-bFGF hydrogels at 48 hours. Scale bar: 200 *μ*m. (d) Quantification of live cells in HP and HP-bFGF hydrogels by Image J. Data were displayed in mean ± standard error from 3 rats in each group. ^∗^ *P* < 0.05, ^∗∗^ *P* < 0.01 versus HP group.

All data were presented in mean ± standard error. Independent samples *t*-test was used in Figures 1(b), 2(b), and 2(d). Otherwise, one-way ANOVA was used. *P* < 0.05 was considered as statistically significant. Statistical analysis was performed using SPSS 19.0 (SPSS, Chicago, IL).

## 3. Results

### 3.1. DPSCs Culture and Identification

**Figure 3:**
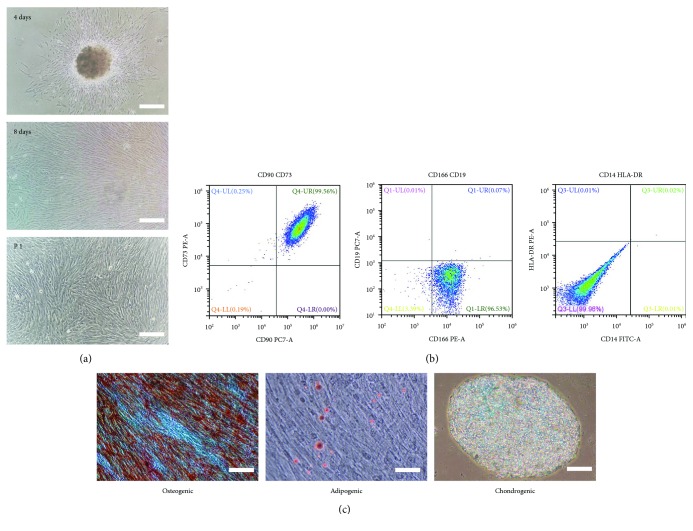
Isolation, culture, and identification of DPSCs. (a) DPSCs culture at day 4, day 8, and the first passage. Scale bar: 200 *μ*m. (b) The expression of surface markers of DPSCs. (c) The osteogenic/adipogenic/chondrogenic differentiation potential of DPSCs. Scale bar: 100 *μ*m.

DPSCs were one kind of MSCs and possessed the properties of MSCs such as expressing MSC-like markers and having multidifferentiation potential.  Figure 3(a) showed that DPSCs displayed a typical fibroblast-like morphology and began to proliferate at day 4 and covered the whole T-25 cm^2^ flask at day 8. The first passage of DPSCs (P_1_) also had great proliferation and survival rate. For the identification of DPSCs, flow cytometry and multilineage differentiation were performed to investigate the MSC-like characteristics of DPSCs. As shown in Figure 3(b), the results of flow cytometric analysis indicated that DPSCs positively expressed MSC-like phenotypic markers, for instance, CD73, CD90, and CD166, but negatively expressed the surface antigen of hematopoietic stem cells such as CD14, CD19, and HLA-DR. As a result of multilineage differentiation (Figure 3(c)), DPSCs had the ability to form mineralized nodules with alizarin red S staining in osteogenic inductive medium, and lipid droplets were observed and stained by oil red O in adipogenic differentiation. Moreover, DPSCs also showed positive results in terms of chondrogenic differentiation with Alcian blue staining.

For neurogenic differentiation, DPSCs were determined by the expression of neural surface markers such as Nestin, NeuN, GFAP, and *β*-tubulin III. The results suggested that DPSCs were positive for Nestin, NeuN, GFAP, and *β*-tubulin III (Figure 1(a)). There was significant difference between the control group and the neurogenic-induced group with the fluorescence intensity of *β*-tubulin III (^∗^ *P* < 0.05), Nestin, and GFAP (^∗∗^ *P* < 0.01), but NeuN showed no difference between the groups (Figure 1(b)).

### 3.2. bFGF Promoted the Proliferation of DPSCs In Vitro

**Figure 4:**
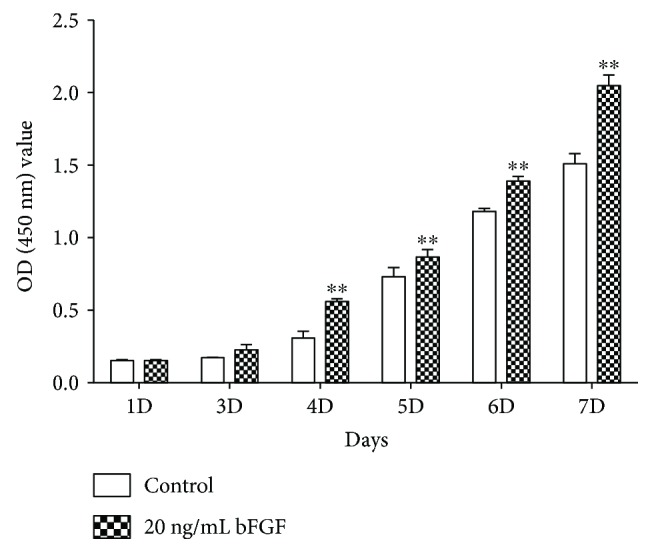
The cell proliferation of DPSCs after bFGF promotion at day 1, 3, 4, 5, 6, and 7. ^∗∗^ *P* < 0.01 versus the control group.

The proliferation of DPSCs cocultured with bFGF in vitro was evaluated by CCK-8 assay (Figure 4). The results showed that the cells were growing up from day 1 to day 7 in both bFGF and control groups. The viability of DPSCs demonstrated no significant difference between control group and bFGF group at day 1 and day 3. However, from day 4 to day 7, the cell proliferation of bFGF group was much higher than that of control group. On the fourth day, the cellular proliferation rate of bFGF group reached the highest level compared to the rest groups.

### 3.3. Morphology of HP and HP-bFGF Hydrogels and Cytocompatibility of Hydrogels with DPSCs

The micromorphology of hydrogels and cytocompatibility of hydrogels with DPSCs were evaluated by SEM and Live/Dead assay, respectively. In Figure 2(a), SEM images of HP and HP-bFGF hydrogels showed porous structure, and the inner pores of hydrogels were interconnected. Meanwhile, the pore size of HP-bFGF hydrogel was smaller than that of HP hydrogel yet more uniform. And the number of pores in HP-bFGF hydrogel was also higher than that of in HP hydrogel (Figure 2(b)). As shown in Figure 2(c), Live/Dead assay results indicated that hydrogels had good cytocompatibility with DPSCs, and numerous DPSCs were stained in green, which were regarded as alive. In addition, the number of viable DPSCs in HP-bFGF hydrogel was more than that of in HP hydrogel (Figure 2(d)).

### 3.4. DPSCs Combined with bFGF Enhanced Motor and Sensory Functional Recovery after SCI

**Figure 5:**
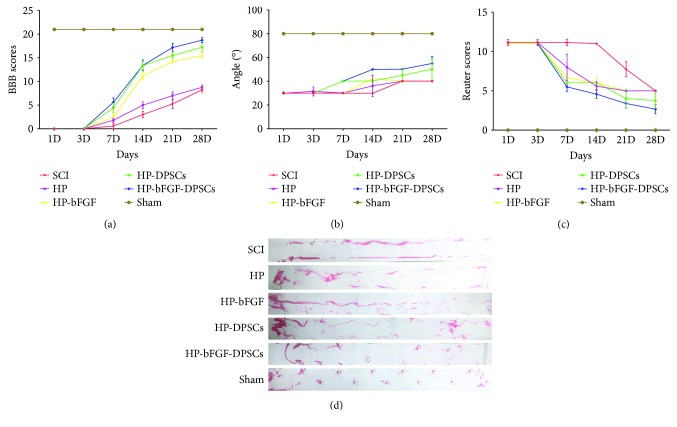
Motor and sensory functional recovery after SCI. (a) The BBB locomotion scores of different groups. (b) The inclined plane test scores of different groups. (c) The Reuter scores of different groups. (d) Footprint analysis of different groups.

The motor and sensory functional recovery after SCI were evaluated by BBB rating scale, inclined plane test, footprint test, and Reuter rating scale, respectively. The hind legs of all animals lost functions and had no movement immediately after SCI. According to BBB scores, inclined plane test scores, and Reuter scores (Figures 5(a), (b), and (c)), the function of hind legs in all experimental groups had no improvement at day 1. From day 3 to day 28, motor functional scores of BBB and inclined plane test were gradually increasing, and decrease of the sensory functional scores of Reuter was observed, which indicated the recovery of sensory function. The functional recovery of hind legs was: HP-bFGF-DPSCs group > HP-DPSCs group > HP-bFGF group > HP group > SCI group. HP-bFGF-DPSCs group showed the strongest beneficial impact on the motor and sensory functional recovery after SCI and had significant difference compared to those of the other experimental groups at day 21 or before (Supplementary Table 1). Meanwhile, the promotion of functional recovery after SCI had no significant difference between HP-bFGF-DPSC group and HP-DPSCs group and showed similar effect at day 28. As shown in footprint test (Figure 5(d)), the results echoed experiment observations of BBB, inclined plane, and Reuter tests, suggesting that HP-bFGF-DPSCs group had the best effect on the functional restoration of hind leg at day 28. The animals in HP-bFGF-DPSCs group showed coordinated crawling with tails raised up, similar to what being observed in the sham control group. On contrast, animals were paralyzed and dragging hind legs in the SCI and HP groups.

### 3.5. Protein Expression of Apoptotic-Related Factors and Neuronal Markers after SCI

**Figure 6:**
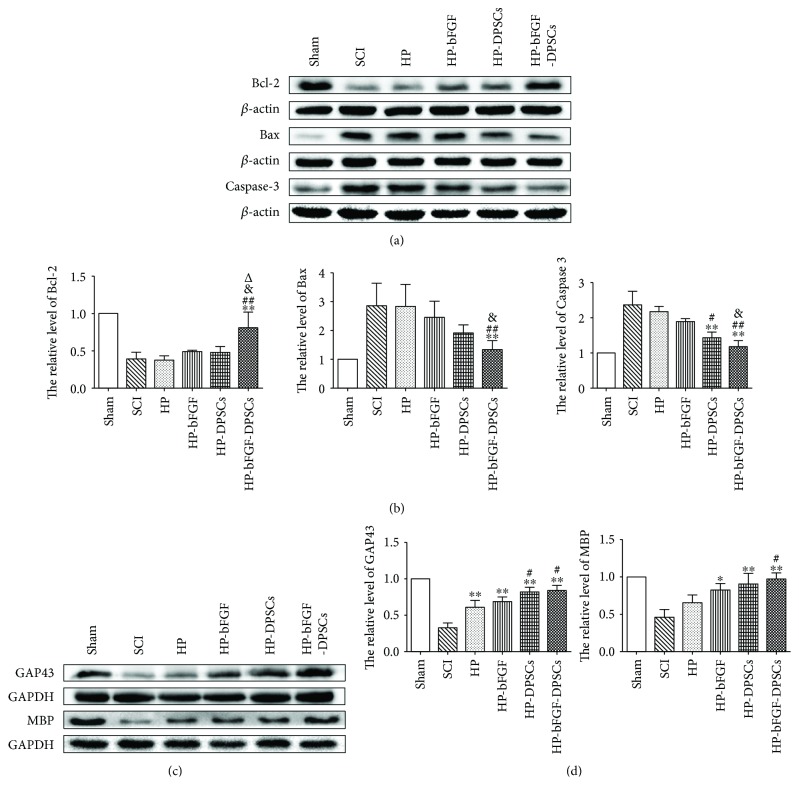
The protein expression of apoptotic-related factors and neuronal markers after SCI. (a) The expressions of Bcl-2, Bax, and Caspase-3 at day 7 after SCI. (b) Quantification of Bcl-2, Bax, and Caspase-3 expression levels. (c) The expressions of MBP and GAP43 at day 21 after SCI. (d) Quantification of MBP and GAP43 expression levels. ^∗^ *P* < 0.05, ^∗∗^ *P* < 0.01 versus SCI group; ^#^ *P* < 0.05, ^##^ *P* < 0.01 versus HP group; ^&^ *P* < 0.05 versus HP-bFGF group; ^Δ^ *P* < 0.05 versus HP-DPSCs group.

In this work, the protein expression of apoptotic-related factors (e.g., Bax, Bcl-2, and Caspase-3) and neuronal markers (e.g., MBP and GAP-43) were detected by western blotting at day 7 and 21, respectively. In all experimental groups, the results showed that the proteins of Bax (proapoptotic factor) and Caspase-3 (the main apoptotic protein) were expressed the most in SCI model group but the least in HP-bFGF-DPSC group which was close to those of the control group. Remarkably, the antiapoptotic factor of Bcl-2 presented the opposite results in the protein expression profile in aforementioned groups (Figure 6(a)). Furthermore, HP-DPSC group also displayed higher expression of Bcl-2 and lower expression of Bax and Caspase-3, similar to those of HP-bFGF-DPSC group. The results of Bcl-2, Bax, and Caspase-3 expression also indicated that HP-bFGF-DPSC group had significant differences compared to the other experimental groups excluding HP-DPSC group (Figure 6(b)). As shown in Figure 6(c), the protein expression levels of MBP and GAP-43 in all experimental groups were HP-bFGF-DPSC group > HP-DPSC group > HP-bFGF group > HP group > SCI group. Almost reaching to a comparable level as the control group, HP-bFGF-DPSC group showed the highest protein expression, followed by HP-DPSC group, whereas the SCI model group had minimum protein expression (Figure 6(d)). Taken together, transplantation of HP hydrogel possessing DPSCs and bFGF could prevent apoptosis and promote new neuron regeneration at both early and later postoperative stages of SCI.

### 3.6. MRI, Histology Evaluation, and Immunohistochemistry

**Figure 7:**
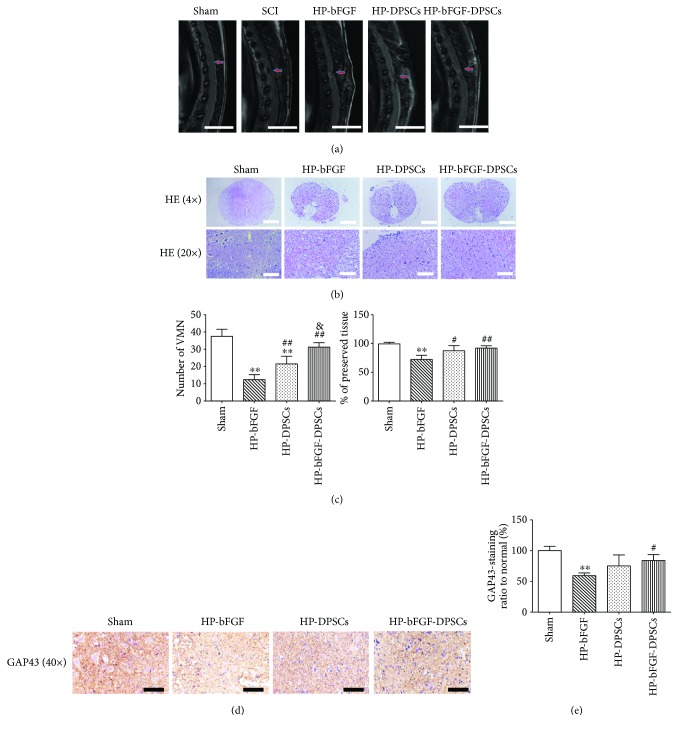
The analyses of MRI, histology evaluation, and immunohistochemistry. (a) MRI images of HP-bFGF, HP-DPSC, and HP-bFGF-DPSC groups at day 28, and SCI modeling stage at 6 hours. Arrows indicated the segmental SCI. Scale bar: 1 cm. (b) Representative images of HE staining at day 28. Scale bar: 500 *μ*m (4×) and 100 *μ*m (20×). (c) Quantification of the number of ventral motor neurons (VMNs) and the percent of preserved tissue in the gray matter of spinal cord. (d) Representative images of GAP43 from immunohistochemisty. Scale bar: 50 *μ*m. (e) Quantification of the GAP43 positive staining ratio to normal in spinal cord. The quantification results obtained by Image J. Data were presented as mean ± standard error from 3 rats in each group. ^∗∗^ *P* < 0.01 versus sham group; ^#^ *P* < 0.05, ^##^ *P* < 0.01 versus HP-bFGF group; ^&^ *P* < 0.05 versus HP-DPSC group.

According to the results of functional recovery analysis and western blotting, the order of effect on neuronal repair and regeneration after SCI in all experimental groups was HP-bFGF-DPSC group > HP-DPSCs group > HP-bFGF group > HP group > SCI model group. Therefore, we chose experimental groups of HP-bFGF-DPSCs, HP-DPSCs, and HP-bFGF, which had better impact on rehabilitation of neurons after SCI, to perform the following experimental analyses: MRI, histology evaluation and immunohistochemistry. According to the MRI results, spinal cord repair and regeneration after SCI could be easily observed. In Figure 7(a), some defects were shown on injured area of spinal cord in HP-bFGF and HP-DPSC groups, which became smaller than that of at the SCI modeling stage. Almost identical to control group, defect was hardly seen on injured area of spinal cord in HP-bFGF-DPSC group, suggesting great tissue restoration had occurred. This indicated that HP-bFGF-DPSCs had the ability to support neuron regeneration and tissue repair after SCI. As shown in Figure 7(b), HE staining suggested that the structure of gray and white matter had been damaged in the experimental groups. Compared to the control group, large-scale destruction of gray matter was observed in HP-bFGF group, yet ventral motor neurons (VMNs) and a few blood vessel regeneration were observed in the damaged areas. In HP-bFGF-DPSC group, abundant blood vessels and VMNs were formed and the scale of the damaged areas was decreased, indicating good effects on tissue repair and regeneration. Quantification of the number of ventral motor neurons (VMNs) and the percent of preserved tissue in the gray matter of spinal cord depicted that HP-bFGF-DPSCs group had similar amount of tissue compared to the control group and more than those of the HP-DPSC group (Figure 7(c)).

According to the immunohistochemical staining of GAP-43, positive expressions were frequently observed in the experimental groups (Figure 7(d)). The intensity of GAP-43 positive regions was HP-bFGF-DPSC group > HP-DPSC group > HP-bFGF group. And the difference between HP-bFGF-DPSC group and control group was statistically insignificant (Figure 7(e)). The results were consistent with previous data, indicating that the combination of HP, DPSCs, and bFGF had provided a promising strategy and beneficial effect on promoting the neuron regeneration and tissue repair after SCI.

## 4. Discussion

Spinal cord injury (SCI), accompanying with motor and sensory dysfunction and disability, which is often caused by traumatic damages, leads to an increase in the socioeconomical costs and compromised quality of life of the injured [1]. It is very difficult to promote neuronal repair and regeneration after SCI because of limitation in necessary precursor cells and secretion of inflammatory cytokines [4]. Dental pulp stem cells (DPSCs) originate from cranial neural crest and display neural-related characteristics including (1) expression of neural-related markers without preinduced differentiation [6, 42]; (2) possession of MSC-like biological properties; and (3) having great capability to differentiate into neuron-like cells and to secrete numerous neurotrophic factors (NFs) in order to provide functional neurons and neuroprotection promoting nerve growth and regeneration [6, 9, 12, 14]. Meanwhile, DPSCs also have vascularization and immunomodulatory properties to enhance blood flow and to improve neural regeneration, respectively [43, 44]. In this work, DPSCs showed the typical MSC-like morphology, for example, fibroblastic and spindle shape and expressed the neural-related markers, for example, Nestin, NeuN, GFAP, and *β*-tubulin III. After differentiation, DPSCs demonstrated an enhanced expression of neural markers and had significant difference in expression of *β*-tubulin III (^∗^ *P* < 0.05), Nestin, and GFAP (^∗∗^ *P* < 0.01) (Figures 1 and 3(a)). The results suggested that DPSCs could be a promising cell source for the treatment of defects in nerve system such as SCI. Although DPSCs have innate advantages in the therapy of SCI compared to stem cells from other sources, for example, good availability, low immunogenicity, and noninvasiveness, it is unlikely to restore the motor and sensory function of SCI using a single DPSCs strategy because the cellular retention and survival rate around injured site cannot be guaranteed. A tissue-engineered construct containing cells, growth factors, and scaffolds could provide a combined strategy promoting axon and neuron repair and regeneration after SCI.

Human basic fibroblast growth factor (bFGF), a growth-promoting stimulus, displays multiple biological functions such as promoting cell proliferation, cell differentiation, and self-renewal [45, 46]. In our study, in vitro studies have been confirmed that bFGF could provide DPSCs beneficial effect on survival and proliferation. Compared to control group, cell proliferation rate of bFGF group reached the highest level at the fourth day and gradually decreased from day 5 to day 7. This phenomenon of decrease could be largely attributed to the fact that DPSCs were approximately 90%–100% confluence around day 4, and the bottom of culture dish was completely covered with monolayer cells. The continuation of cell culture after a full confluence had a negative effect on cell growth for the existed inhibition of cellular contact, which resulted in the drop of cell proliferation rate in bFGF group in day 5–7 [47]. However, the cell proliferation of bFGF group was always higher than that of control group in in vitro assay. Moreover, studies indicated that bFGF had the capability to promote axon regeneration, to provide neuroprotection, and to improve behavioral effects in vivo after SCI [19, 21]. Other experiments also showed that bFGF could enhance the survival of neurons and prohibit apoptosis in injured site of SCI [48, 49]. As a macromolecule protein that has very short half-life, bFGF is usually cleaned from the tissue very fast through enzymatic degradation and diffusion [22]. Therefore, new delivery system such as nanoliposomes and biomaterial scaffolds can provide promising strategies to overcome these limitations [30, 33].

Hydrogel has been considered as an attractive biomaterial scaffold for tissue engineering owing to its unique structure to mimic the natural extracellular matrix, to control release bioactive molecules, and to accommodate seeded cells [23]. 3D network of hydrogel scaffold is suitable for cell adhesion, growth, and proliferation. Meanwhile, hydrogel possesses the biomimetic environment that can be used to load and encapsulate biological macromolecules preventing from rapid diffusion and enzymatic degradation [30, 33, 34]. Therefore, hydrogel can act as a substitute to extracellular matrix to provide an engineered scaffold for tissue regeneration after SCI [30]. In this study, we developed HP thermosensitive hydrogel which was nontoxic and had 3D porous structure. The HP hydrogel was prepared from heparin and poloxamer and had a high affinity with growth factors through the heparin-SH group. According to our previous studies [29], HP hydrogel had the ability to provide protective agents for loading and delivering biological macromolecules such as aFGF and NGF, promoting axon regeneration and new blood vessel formation in injured site after SCI. Moreover, HP had good biocompatibility with neural cell lines such as PC12 in vitro. Thus, in this research, HP hydrogel had been used as a scaffold to load and deliver bFGF and DPSCs for in situ administration after SCI because of its 3D network structure, good biocompatibility, and high affinity.

Live/Dead assay was performed to investigate the cytocompatibility of HP hydrogel with DPSCs in vitro in our study. Because of its nontoxicity and mild nature, HP hydrogel has been shown to possess good compatibility with DPSCs. The effects of transplanted HP hydrogel containing bFGF and DPSCs on axon regeneration and neuron repair after SCI were analyzed by functional recovery tests, Western blot, MRI, HE, and immunohistochemistry. The functional recovery results showed that animals which were treated with HP-bFGF, HP-DPSCs, and HP-bFGF-DPSCs had better outcome than the HP and SCI groups. Meanwhile, the degree of locomotor and sensory recovery of HP-bFGF-DPSC group was the best among the experimental groups, indicating that application of DPSCs combined with bFGF had a stronger impact on restoration and regeneration of neuronal function than bFGF-alone and DPSC-alone applications. Bcl-2 as an antiapoptotic factor as well as Bax and Caspase-3 as pro-apoptotic factors regulate the cellular survival and proliferation. Studies suggested that bFGF could prevent apoptosis by upregulating the expression of Bcl-2 and downregulating the expression of Bax in order to promote the cell proliferation [50, 51]. DPSCs had been found to regulate the expression of Caspase-3 by secreting and producing many immunomodulatory factors [52]. In our study, HP-bFGF-DPSC group demonstrated the highest expression of Bcl-2 and the lowest expression of Bax and Caspase-3, which was consistent with the outcome of functional recovery analysis. Furthermore, the neural-related markers of GAP-43 and MBP also had the highest expression in HP-bFGF-DPSC group.

MRI results were used to reflect the degree of repair in spinal cord after SCI in vivo. HP-bFGF-DPSC showed the best impact on the spinal cord regeneration. The damaged area of HP-bFGF-DPSCs almost disappeared and was replaced by newly regenerated tissue, which was similar to the control group. HE staining showed that HP-bFGF-DPSC group had more newly regenerated cells and blood vessels than HP-bFGF and HP-DPSC group did. And the injured area of HP-bFGF-DPSCs group was the smallest compared to the other experimental groups, similar to the control group. Immunohistochemistry of GAP-43 staining suggested that HP-bFGF-DPSCs group had the most GAP-43 positive expression, indicating that DPSCs combined with bFGF had stronger promotion of neuronal regeneration than single bFGF or DPSCs strategy. Taken together, all results indicated that the combination of HP hydrogel, DPSCs, and bFGF had more impact on neuronal regeneration, functional recovery, and tissue repair than transplanted HP with bFGF-alone or DPSC-alone strategies, which can be a promising strategy to promote neuron regeneration and tissue repair after SCI.

## 5. Conclusions

This study for the first time identified an optimal combination of scaffold, cell, and growth factor for neuronal regeneration as well as functional recovery after SCI. Our results clearly demonstrated that transplanted HP hydrogel containing DPSCs and bFGF resulted in remarkably beneficial effects on the treatment of SCI. Therefore, the study provided a novel therapeutic strategy for unmet clinical needs in neuron repair, function restoration, and tissue regeneration after SCI.

## Supplementary Materials

Supplementary Materials The supplementary material provides the results of pairwise statistical analysis of functional behavioral score between groups. BBB scores: ^∗^ *P* < 0.05, ^∗∗^ *P* < 0.01, ^∗∗∗^ *P* < 0.001; Reuter scores: ^#^ *P* < 0.05, ^##^ *P* < 0.01, ^###^ *P* < 0.001; Angle scores: ^&^ *P* < 0.05, ^&&^ *P* < 0.01, ^&&&^ *P* < 0.001.
