# Correction to: “Effects of Transplanted Heparin‐Poloxamer Hydrogel Combining Dental Pulp Stem Cells and bFGF on Spinal Cord Injury Repair”

**DOI:** 10.1155/sci/9817341

**Published:** 2026-10-23

L. Luo, A. A. Albashari, X. Wang, et al., “Effects of Transplanted Heparin‐Poloxamer Hydrogel Combining Dental Pulp Stem Cells and bFGF on Spinal Cord Injury Repair,” *Stem Cells International* (2018): 1–13, https://doi.org/10.1155/2018/2398521.

Following publication, the authors identified errors in Figures 1a and 3b, which were introduced during the data processing stage.

1. In Figure 1a (double‐staining of Nestin and β‐tubulin III), the DAPI‐stained nucleus image was inadvertently duplicated and incorrectly overlaid in the published version.
2. In Figure 3b, the flow cytometry scatter plot was inadvertently presented without proper compensation, resulting in an incorrect representation of the data.

**Figure 1:**
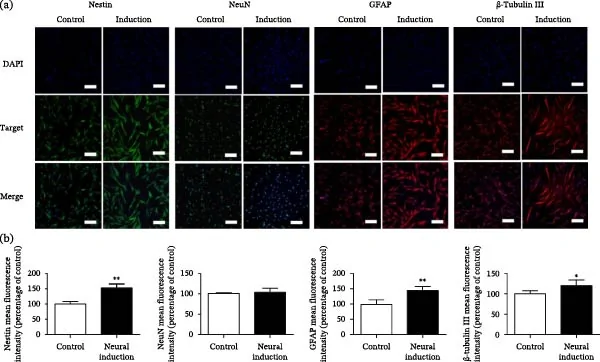
The neurogenic differentiation potential of DPSCs. (a) The expressions of Nestin, NeuN, GFAP, and β‐tubulin III. Scale bar: 200 μm. (b) Quantification of the fluorescence intensity of Nestin, NeuN, GFAP, and β‐tubulin III.  ^∗^ *p* < 0.05,  ^∗∗^ *p* < 0.01 versus the control group.

**Figure 3:**
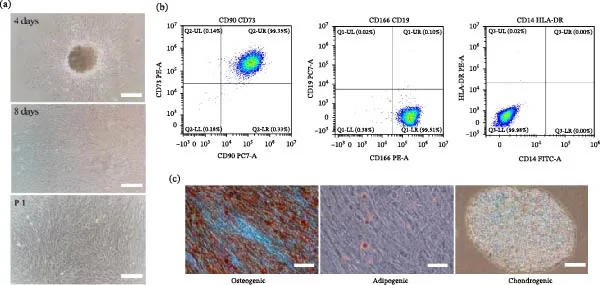
Isolation, culture, and identification of DPSCs. (a) DPSCs culture at day 4, day 8, and the first passage. Scale bar: 200 μm. (b) The expression of surface markers of DPSCs. (c) The osteogenic/adipogenic/chondrogenic differentiation potential of DPSCs. Scale bar: 100 μm.

The authors have provided the correct images to accurately reflect the underlying data. This correction does not affect the interpretation of the results or the conclusions of the article.

We apologize for these errors.

