# Supplementary material for: Effects of Transplanted Heparin-Poloxamer Hydrogel Combining Dental Pulp Stem Cells and bFGF on Spinal Cord Injury Repair
Source: Stem Cells Int. 2018 Mar 27;2018:2398521. doi: 10.1155/2018/2398521 (PMC5892218; doi:10.1155/2018/2398521)
Supplement: Supplementary Materials — The supplementary material provides the results of pairwise statistical analysis of functional behavioral score between groups. BBB scores: ∗ P < 0.05, ∗∗ P < 0.01, ∗∗∗ P < 0.001; Reuter scores: # P < 0.05, ## P < 0.01, ### P < 0.001; Angle scores: & P < 0.05, && P < 0.01, &&& P < 0.001. [file 2398521.f1.pdf]

Table Supplementary 1 (S1): Results of pairwise statistical analysis of functional behavioral score between groups

Day	Group	SCI	HP	HP-bFGF	HP-DPSCs	HP-bFGF- DPSCs	sham
1D	SCI						***
	HP						***
	HP-bFGF						***
	HP-DPSCs						***
	HP-bFGF- DPSCs						***
	sham	###	###	###	###	###	
1D	SCI						
	HP						
	HP-bFGF						
	HP-DPSCs						
	HP-bFGF- DPSCs						
	sham	&&&	&&&	&&&	&&&	&&&	
3D	SCI						***
	HP						***
	HP-bFGF						***
	HP-DPSCs						***
	HP-bFGF- DPSCs						***
	sham	###	###	###	###	###	
3D	SCI						
	HP						
	HP-bFGF						
	HP-DPSCs						
	HP-bFGF- DPSCs						
	sham	&&&	&&&	&&&	&&&	&&&	
7D	SCI			**	***	***	***
	HP	###			***	***	***
	HP-bFGF	###			***	***	***
	HP-DPSCs	###	###				***
	HP-bFGF- DPSCs	###	###				
	sham	###	###	###	###	###	
7D	SCI						
	HP						
	HP-bFGF						
	HP-DPSCs						
	HP-bFGF- DPSCs	&&&	&&&	&&&			
	sham	&&&	&&&	&&&			
14D	SCI		*	***	***	***	***
	HP	###		***	***	***	***
	HP-bFGF	###			**	**	***
	HP-DPSCs	###					***
	HP-bFGF- DPSCs	###					
	sham	###	###	###	###	###	

14D	SCI						
	HP						
	HP-bFGF	&					
	HP-DPSCs	&&&					
	HP-bFGF- DPSCs	&&&			&&&		
	sham	&&&	&&&	&&&	&&&	&&&	
21D	SCI		*	***	***	***	***
	HP			***	***	***	***
	HP-bFGF					***	***
	HP-DPSCs	###				*	***
	HP-bFGF- DPSCs	###					***
	sham	###	###	###	###	###	
21D	SCI						
	HP						
	HP-bFGF						
	HP-DPSCs						
	HP-bFGF- DPSCs	&&&	&&&	&&&			
	sham	&&&	&&&	&&&	&&&	&&&	
28D	SCI			***	***	***	***
	HP			***	***	***	***
	HP-bFGF					***	***
	HP-DPSCs						*
	HP-bFGF- DPSCs						*
	sham	###	###	###	###	###	
28D	SCI						
	HP						
	HP-bFGF						
	HP-DPSCs						
	HP-bFGF- DPSCs						
	sham	&&&	&&&	&&&	&&&	&&&	

BBB scores: $^{*}p < 0.05$, $^{**}p < 0.01$, $^{***}p < 0.001$; Reuter scores: $^{\#}p < 0.05$, $^{\#\#}p < 0.01$, $^{\#\#\#}p < 0.001$; Angle scores: $^{\&}p < 0.05$, $^{\&\&}p < 0.01$, $^{\&\&\&}p < 0.001$
